# Altered plasma *p*‐tau in presymptomatic and symptomatic neuronal intranuclear inclusion disease

**DOI:** 10.1002/alz70856_100133

**Published:** 2025-12-25

**Authors:** Sizhe Zhang, Bin Jiao, Yan Zeng, Beisha Tang, Yun Tian, Lu Shen

**Affiliations:** ^1^ Xiangya Hospital, Central South University, Changsha, Hunan, China

## Abstract

**Background:**

Neuronal intranuclear inclusion disease (NIID) caused by GGC repeat expansions in *NOTCH2NLC*, is a neurodegenerative disease characterized by widespread intranuclear inclusions, and is frequently involved with cognitive impairment. Limited studies have focused on the biomarkers alteration in patients with NIID and presymptomatic NIID (preNIID) individuals. Comparison between NIID and Alzheimer's disease (AD) is lacking.

**Method:**

Two cohorts were enrolled. Cohort 1 with 344 participants included 87 patients with NIIID, 147 patients with AD, and 110 healthy controls (HCs). Cohort 2 included 26 preNIID individuals and 26 matched HCs. Eight widely used AD plasma biomarkers including Amyloid‐β (Aβ) 40, Aβ42, neurofilament light (NfL), α‐synuclein (α‐syn), phosphorylated tau protein 181 (*p*‐tau181), *p*‐tau217, *p*‐tau231 and glial fibrillary acidic protein (GFAP) were detected. Comprehensive neuropsychological scores and MRI measures were collected.

**Result:**

NfL, α‐syn, *p*‐tau181, *p*‐tau217, *p*‐tau231 and GFAP levels were significantly higher in NIID than HCs, and the α‐syn, *p*‐tau181, *p*‐tau217 and *p*‐tau231 levels were comparable between NIID and AD. NfL level in NIID was higher than AD, and GFAP in NIID was lower compared with AD. Interestingly, *p*‐tau217 showed the best diagnostic performance in distinguishing NIID from HCs, with an AUC of 0.814. Moreover, the level of *p*‐tau217 was associated with MMSE and FAB scores in dementia‐dominant subtype, and the level of GFAP was correlated to white matter volume. We also observed that multiple *p*‐tau species and GFAP were already elevated in preNIID. Both *p*‐tau217 and GFAP performed well in recognizing preNIID (AUC ∼0.850). Surprisingly, *p*‐tau species, including *p*‐tau217 were difficult in distinguishing NIID from AD.

**Conclusion:**

This study elucidated the biomarkers alteration in NIID comprehensively. The changes of *p*‐tau levels, especially *p*‐tau217, provided a new aspect for the demonstration of NIID pathophysiology, and early alteration of *p*‐tau species and GFAP in preNIID further validated their potential value as early biomarkers of NIID. Impressively, *p*‐tau species could not distinguish NIID from AD.

